# Validation of Automatically Generated Global and Regional Cropland Data Sets: The Case of Tanzania

**DOI:** 10.3390/rs9080815

**Published:** 2017-08-09

**Authors:** Juan Carlos Laso Bayas, Linda See, Christoph Perger, Christina Justice, Catherine Nakalembe, Jan Dempewolf, Steffen Fritz

**Affiliations:** 1 Ecosystems Services and Management Program, International Institute for Applied Systems Analysis (IIASA), Laxenburg A-2361, Austria; see@iiasa.ac.at (L.S.); pergerch@iiasa.ac.at (C.P.); fritz@iiasa.ac.at (S.F.); 2 Department of Geographical Sciences, University of Maryland, College Park, Maryland, MD 20742, USA; justicec@umd.edu (C.J.); cnakalem@umd.edu (C.N.); dempewol@umd.edu (J.D.)

**Keywords:** land cover, validation, cropland, Geo-Wiki, agricultural monitoring

## Abstract

There is a need to validate existing global cropland maps since they are used for different purposes including agricultural monitoring and assessment. In this paper we validate three recent global products (ESA-CCI, GlobeLand30, FROM-GC) and one regional product (Tanzania Land Cover 2010 Scheme II) using a validation data set that was collected by students through the Geo-Wiki tool. The ultimate aim was to understand the usefulness of these products for agricultural monitoring. Data were collected wall-to-wall for Kilosa district and for a sample across Tanzania. The results show that the amount of and spatial extent of cropland in the different products differs considerably from 8% to 42% for Tanzania, with similar values for Kilosa district. The agreement of the validation data with the four different products varied between 36% and 54% and highlighted that cropland is overestimated by the ESA-CCI and underestimated by FROM-GC. The validation data were also analyzed for consistency between the student interpreters and also compared with a sample interpreted by five experts for quality assurance. Regarding consistency between the students, there was more than 80% agreement if one difference in cropland category was considered (e.g., between low and medium cropland) while most of the confusion with the experts was also within one category difference. In addition to the validation of current cropland products, the data set collected by the students also has potential value as a training set for improving future cropland products.

## 1 Introduction

To ensure global food security, cropland is regularly monitored by initiatives such as GEOGLAM (Group on Earth Observation’s Global Agricultural Monitoring) [1], CropWatch [2], and the MARS (Monitoring Agricultural Resources) unit of the Joint Research Centre of the European Commission, among others [3]. Agricultural monitoring relies heavily on the use of Earth Observation, e.g., from yield and production estimation to identification of cropping patterns [4], which requires baseline information on cropland as a key input [5]. Many different products now exist and must be validated in order to understand whether they are fit for purpose for agricultural monitoring. In the past, medium to coarse resolution imagery from sensors such as AVHRR, SPOT-VGT, MERIS, and MODIS has been used extensively to map land cover, e.g., [6–8] and cropland, e.g., [1,9,10]. These products are mostly generated using a top down approach, employing automated or semi-automated classification techniques and a training data sample collected from field data, interpretation of satellite or aerial imagery, or both. However, when these products are compared, there are often large spatial disagreements between them, particularly in the cropland class [11], which has led to the production of hybrid or unified cropland products [12,13] in an attempt to improve the overall accuracy compared to individual products, and in terms of representation over space. Higher resolution Landsat imagery has also been employed, e.g., in the production of land cover products that include a cropland class such as FROM-GLC [14], FROM-GC [15], and GlobeLand30 [16], and more recently demonstrated on a small area using Sentinel-2 [17].

Although these resolutions are higher than the more commonly used medium to coarse resolution sensors employed in cropland mapping, they cannot capture very small field sizes, there may be fuzzy field boundaries, and in places such as Africa, there is a spectral and structural similarity between agricultural fields and the surrounding natural vegetation. In the developing world, there are many areas with smallholder agriculture and small field sizes. For example, it is estimated that 84% of farms globally are less than 2 ha in size; this represents around 30–40% of land in Sub-Saharan Africa and Asia [18], where individual fields will be even smaller. According to [19], more than 75% of agricultural output in East Africa alone is produced on farms with an average size of 2.5 ha. Thus, to map cropland in areas dominated by smallholder agriculture, high resolution data (5–30 m) or very high resolution (VHR) satellite or aerial imagery (i.e., with resolutions of2mor less) is required, both for calibration to improve spectral separation and for validation of existing cropland products.

The Geo-Wiki tool [20,21] is an application for collecting land cover classifications at sample locations around the world through visual interpretation of VHR imagery using crowdsourcing, which is a term to denote the outsourcing of micro-tasks to the crowd [22]. Using a bottom up approach for cropland characterization, a number of successful crowdsourcing campaigns have been run to collect data for the generation of hybrid land cover maps, including a global cropland extent map and a global map of field size [12]. In this paper we used a modified offline version of Geo-Wiki and recruited and trained a set of 20 students in Tanzania to undertake the visual interpretation of VHR imagery. This also allowed us to use the local knowledge of people living on the ground. The students collected a sample of data across Tanzania and a wall-to-wall data set for the district of Kilosa to form a reference validation data set. Thus, the aim of this paper is to validate three global and one regional land cover products for cropland, all of which have been produced through automatic classification in order to understand how well the different products capture cropland, both in terms of total area and in their spatial distribution. This will help us to determine how fit for purpose the different data sets are for agricultural monitoring.

In the next section we describe the global land cover and cropland data sets that were used for comparison with the data collected by the students in Tanzania. This is followed by a description of the methods used for validation. The results are then presented, followed by a discussion on the merits and limitations of such an approach. Finally, we consider the suitability of the different products for agricultural monitoring.

## 2 Materials and Methods

### 2.1 Global and Regional Land Cover Maps

The first data set to be validated is a 300 m resolution global land cover map (ESA-CCI), which was produced for the climate change initiative (CCI) of the European Space Agency in the framework of the Copernicus land monitoring service [23]. The ESA-CCI product was developed using imagery from five years, i.e., 2008–2012. Using time series from the MERIS and SPOT-VGT sensors, machine learning and unsupervised classification algorithms were used to classify the spectral characteristics of the composite images, which were then merged with a reference land cover database created from existing land cover maps. Finally, the product was updated with 10 years of land cover produced separately from 2003 to 2012 to create a baseline product for 2010. By using a multi-temporal multi-sensor approach, a more stable baseline land cover product was produced. The ESA-CCI product has 22 different land cover classes, where four classes correspond to cropland or mosaics of cropland. The overall accuracy of the map is reported to be 74.4%. The producer’s accuracy is 80% and 77% for rainfed and irrigated cropland, respectively, while the corresponding user’s accuracy for these two cropland classes is 88% and 92%, respectively [24]. The mosaic cropland classes are not considered further in this paper.

GlobeLand30 is the second global land cover product used in this study, which is a 30 m land cover product produced for the years 2000 and 2010 by the National Geomatics Center in China [16]. Using more than 10,000 scenes from Landsat, a pixel-object-knowledge-based classification approach was used, which involved both pixel-based and segmentation approaches on a per-class basis to identify 10 major classes, one of which is cultivated land. Using MODIS and Landsat time series in a NDVI-learning mixing growth model, a 30 m NDVI time series was used with a supervised classifier to identify potential areas of cropland. The results from the pixel-based approach were then overlain on the potential areas of cropland identified through segmentation and only those objects with greater than 70% cultivated land with regular man-made patterns were then labelled as being in the cultivated land class. The overall area-weighted accuracy of this product is reported to be 79.26 ± 0.2% with a user’s accuracy of 82.8% for cultivated land. The authors do not report a producer’s accuracy for cultivated land as part of their accuracy assessment [16]. Moreover, recent comparisons of GlobeLand30 with authoritative land cover data sets in Germany, Italy, and Scandinavia have shown good overall agreement [25–27].

The third reference data set used was FROM-GC [15], which is an improved 30 m spatial resolution cropland product developed by combining different global land cover maps, and has been produced by Tsinghua University in China (i.e., integrating the FROM-GLC and FROM-GLC-agg global land cover products with the MODIS-based cropland probability map of Pittman et al. [10]). The FROM-GLC global land cover product corresponds to the year 2010; it was produced using 9000 Landsat images from around this period [28] and contains seven higher level land and 26 second level land cover classes. Unlike GlobeLand30, which included a considerable amount of manual input from interpreters, FROM-GLC was produced in a completely automated fashion using a support vector machine-based learning algorithm. The FROM-GLC-agg product is an improved version of FROM-GLC, which includes additional impervious layers, e.g., MODIS urban extent [29]. The overall accuracy is 65.51% [29] while the producer’s and user’s accuracies for cropland are reported to be 66.6% and 57.6%, respectively [15]. The areas of cropland were then calculated and compared to FAO cropland area statistics, resulting in a correlation of 0.97 [15]. However, the results generally showed an underestimation in countries with small cropland and in tropical regions, particularly those in Africa. The region referred to as Middle Africa in the paper, which would include Tanzania, had the highest underestimation in cropland at 37.3%.

The fourth and final land cover data set is the Tanzania Land Cover 2010 Scheme II product, which is part of a regional eastern and southern Africa land cover data set produced by the Regional Centre for Mapping of Resources for Development (RCMRD) and SERVIR [30]. The map was generated for the period 2010 from Landsat thematic mapper (Landsat 5) imagery using the maximum likelihood classification method and has a resolution of 30 m. Additional procedures such as filtering, pixel/cell editing, and density slicing were performed to refine the classification. Accuracy assessment was done using data collected in the field and point interpretation from Landsat imagery. The map has 19 main classes, where two refer to cropland (perennial and annual). The coverage includes nine Eastern and Southern Africa countries: Ethiopia, Botswana, Lesotho, Malawi, Namibia, Rwanda, Tanzania, Uganda, and Zambia. The map’s reported overall accuracy is 77%.

### 2.2 Validation Data Collected by the Trained Students

The data were collected by 20 students who were undergraduates studying in the Department of Agricultural Engineering and Land Planning in the Faculty of Agriculture at Sokoine University of Agriculture in Tanzania. Their subjects included Agricultural Engineering, Irrigation and Water Resources Engineering, Bioprocess and Post-harvest Engineering, and Land Resources Management. Hence they did not have a specific background in GIS or remote sensing but training was provided during a workshop held in Tanzania. Prizes were offered as incentives to participate.

**Figure 1:**
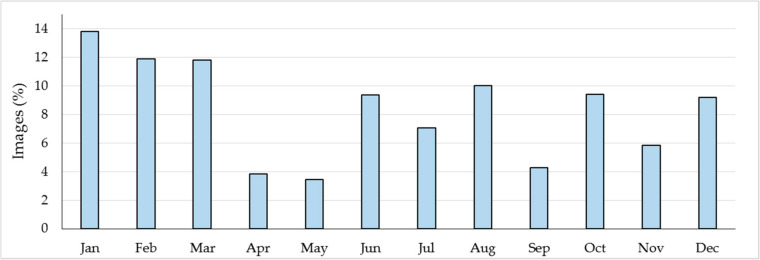
Monthly distribution of very high resolution (VHR) imagery that was used during the data collection campaign.

In total, the students visually interpreted 25,943 VHR satellite images spanning the period 2005 to 2014. The distribution of images across the year by percentage is shown in Figure 1.

**Figure 2:**
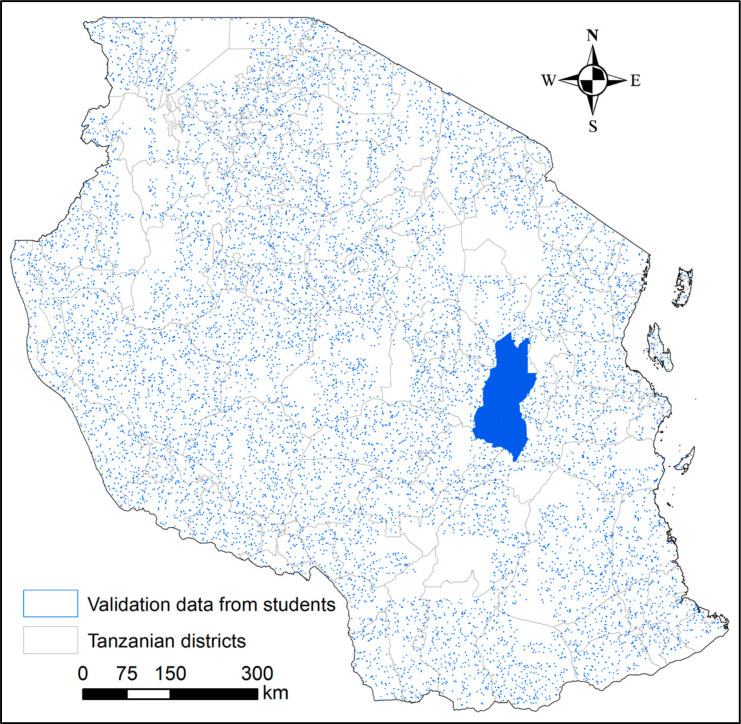
Location and distribution of data collected across Tanzania with full coverage (wall-to-wall) in the Kilosa district.

**Figure 3:**
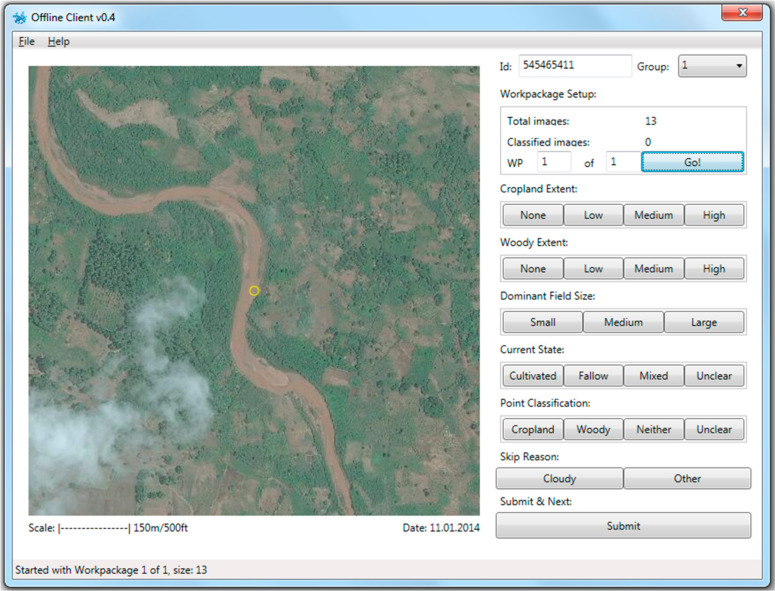
The Geo-Wiki offline interface for collecting data on cropland and woodland extent.

A total of 15,383 images covered the Kilosa district wall-to-wall while the rest were randomly distributed across the rest of Tanzania (Figure 2). The campaign used a customized offline branch of the Geo-Wiki application, as shown in Figure 3, to visually interpret 1 km × 1 km VHR images. These RGB images, comprising a mosaic of mainly WorldView-2 imagery, were provided by Digital Globe through their viewing service, which is a Web Map Service (WMS). A resolution of 1 km was chosen because enough detail is visible in the imagery to be able to distinguish between cropland and non-cropland. Although 300 m would have been also possible, this would increase the sample size by almost 10 times and not much additional detail would be visible. Hence 1 km is a compromise between detail and efficiency in data collection. Data collection at 30 m would have required too many samples.

In Figure 3, the image displayed on the left of the screen shows the area to be classified; below the image is a scale (in meters and feet), to help determine the field size, and the image date. Each user entered a personal unique identification name or code. Set up by work packages, the number of images per work package was indicated at the bottom so that progress could be monitored. For each image, the students were asked to determine the:

- Cropland Extent (None/Low/Medium/High)
- Woody Extent (None/Low/Medium/High)
- Dominant Field Size (Small/Medium/Large)
- Current State (Cultivated/Fallow/Mixed/Unclear)
- Point Classification (Cropland/Woody/Neither/Unclear)

Cropland is defined according to the FAO definition of arable and permanent crops, including fallow land of less than five years [31]. The thresholds between low, medium, and high cropland and woody extent were roughly set at one-third (between low and medium) and two-thirds (between medium and high) to aid in visual interpretation. If the image was difficult to classify or if there were clouds present, they could then skip the grid square and move on to the next one.

The campaign was completed when each grid square was classified by each student, and where possible, at least twice, allowing the majority of images to have more than one evaluation by different participants in order to cross check the outputs. The result was a wall-to-wall cropland and woodland extent map for Kilosa. All the data sets used in the study are shown in Table 1.

To determine the variation across students in the validation data set, three data metrics were used for comparison, i.e., the minimum, the median, and the maximum cropland values across all students for the same image. Table 2 shows averages per image for the lowest, mean, and highest values for cropland and woodland extent from 22,190 images across Tanzania where there was more than one student evaluating the image (in percent). Moreover, for any given image, the average standard deviation between students was around 15% for cropland extent and 14% for woody extent.

**Table 1:** Data sets used in this study. * denotes frames containing Digital Globe very high resolution (VHR) imagery.

Data Set	Reach	Resolution	Imagery Timespan
ESA-CCI	Global	300 m	2008–2012 (2010 baseline)
GlobeLand30	Global	30 m	2008–2011 (2010 baseline)
FROM-GC	Global	30 m	2010
RCMRD	Multi-national	30 m	2010
Validation data from the students	National	1 × 1 km frame *	2005–2014

**Table 2:** Descriptive statistics for the data collected through the Geo-Wiki interface for cropland and woody extent in percentage. Images with only one validation are excluded (N = 22,190).

Variable	Average Lowest	Average Mean	Average Highest
Cropland extent	13.6	25.8	38.5
Woody extent	42.8	54.3	65.6

### 2.3 Comparison of the Data Sets

#### 2.3.1 Pre-Processing

Prior to comparison, some pre-processing of the data sets was required. The first step involved reclassifying the four land cover products so that the corresponding cropland classes were set to 1 and all other classes were set to 0. Table 3 shows the different cropland classes in the four data sets, their corresponding definitions and LCCS (Land Cover Classification System) labels, codes and levels. Additionally, Table 4 provides the LCCS labels and aggregated classes for each of the data sets used in the study for comparison purposes. In the case of the ESA-CCI, there were two cropland classes, one cultivated land class in GlobeLand30, four in FROM-GC and two in the RCMRD data set. Note that the mosaic classes in ESA-CCI were not considered.

**Table 3:** Cropland classes in each data set and their corresponding LCCS (Land Cover Classification System) notations.

Data Sets	Class	Definition	LCCS Label^*^	LCCS Code	LCCS Level
ESA-CCI	10	Cropland, rainfed	Rainfed shrub crops	11494	A2XXXXXXD1
			Rainfed tree crops	11490	A1XXXXXXD1
			Rainfed herbaceous crops	11498	A3XXXXXXD1
	20	Cropland, irrigated or post-flooding	Irrigated tree crops	11491	A1XXXXXXD3
			Irrigated shrub crops	11495	A2XXXXXXD3
			Irrigated herbaceous crops	11500	A3XXXXXXD3
			Post-flooding cultivation of herbaceous crops	11499	A3XXXXXXD2
GlobeLand30	10	Cultivated land: Lands used for agriculture, horticulture and gardens, including paddy fields, irrigated and dry farmland, vegetation and fruit gardens, etc.	Rainfed shrub crops	11494	A2XXXXXXD1
			Rainfed tree crops	11490	A1XXXXXXD1
			Rainfed herbaceous crops	11498	A3XXXXXXD1
			Irrigated tree crops	11491	A1XXXXXXD3
			Irrigated shrub crops	11495	A2XXXXXXD3
			Irrigated herbaceous crops	11500	A3XXXXXXD3
			Post-flooding cultivation of herbaceous crops	11499	A3XXXXXXD2
FROM-GC	11	Crop-Rice	Irrigated herbaceous crops	11500	A3XXXXXXD3
	12	Crop-Greenhouse	NA^†^	NA	NA
	13	Crop-Other	Herbaceous crop(s)	10025	A3
			Shrub crop(s)	10013	A2
	94	Bare-Cropland	Bare soil and/or other unconsolidated material(s) Herbaceous croplands	6005	A5
				10025	A3
RCMRD	41	Perennial Cropland	Rainfed shrub crops	11494	A2XXXXXXD1
			Rainfed tree crops	11490	A1XXXXXXD1
			Irrigated tree crops	11491	A1XXXXXXD3
			Irrigated shrub crops	11495	A2XXXXXXD3
	42	Annual Cropland	Rainfed herbaceous crops	11498	A3XXXXXXD1
			Irrigated herbaceous crops	11500	A3XXXXXXD3
			Post-flooding cultivation of herbaceous crops	11499	A3XXXXXXD2
Validation data from the students	Scale: 1-2-3-4	1: No cropland, 2: Low cropland, 3: Medium cropland and 4: High cropland	Rainfed shrub crops	11494	A2XXXXXXD1
			Rainfed tree crops	11490	A1XXXXXXD1
			Rainfed herbaceous crops	11498	A3XXXXXXD1
			Irrigated tree crops	11491	A1XXXXXXD3
			Irrigated shrub crops	11495	A2XXXXXXD3
			Irrigated herbaceous crops	11500	A3XXXXXXD3
			Post-flooding cultivation of herbaceous crops	11499	A3XXXXXXD2

* LCCS labels, codes and levels were taken from the ESA CCI Manual [32] except for FROM-GC, which were taken from Gong et al. [28].

† Greenhouse agriculture is hard to define under the LCCS system [28]. The class Crop-Greenhouse is included in this analysis as the overall crop proportion in the FROM-GC data set is very low.

**Table 4:** LCCS labels and their correspondence in each of the data sets used in the study. A check mark (✓) indicates correspondence with the LCCS label while an X (✗) denotes absence.

LCCS-Label	Aggregated Class	ESA-CCI	GlobeLand30	FROM-GC	RCMRD	Validation Data from the Students
Rainfed herbaceous crops Irrigated herbaceous crops Post-flooding cultivation of herbaceous crops Herbaceous croplands	Herbaceous crops	✓	✓	✓	✓	✓
Rainfed shrub crops Irrigated shrub crops	Shrub Crops	✓	✓	✓	✓	✓
Rainfed tree crops Irrigated tree crops	Tree Crops	✓	✓	✗	✓	✓
Bare soil and/or other unconsolidated material(s)	✗	✗	✓	✗	✗

The validation data set produced by the students contains four possible classes, i.e., 1 = no cropland, 2 = low cropland, 3 = medium cropland, and 4 = high cropland. These values were linearly transformed into values between 0 and 1.

**Figure 4:**
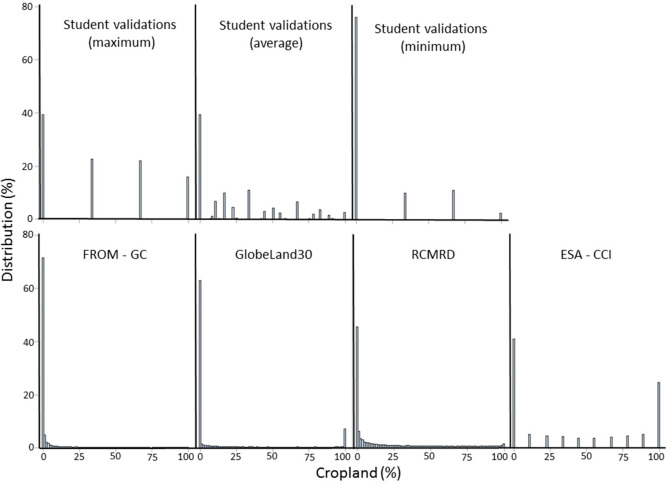
Histograms of all data sets compared showing percentage cropland in the 1 × 1 km areas.

The information from each of these four products was aggregated to match the grid of the validation data set using ArcGIS zonal statistics to produce cropland proportions in each 1 km grid cell. The values from these four products were then extracted for each of the 25,943 1 × 1 km images interpreted by the students. From this sample, 22,190 had at least two interpretations from two different students and hence averages could be calculated across all evaluations. The resulting histograms are shown in Figure 4. Both the validation data and the ESA-CCI show a range of cropland proportions while the other data sets are heavily skewed towards lower proportions of cropland.

#### 2.3.2 Statistical Comparison of Overall Cropland

A generalized linear model with a binomial distribution and a logit link with a Laplace estimation was used to compare the proportion of cropland generated by each data set. The SAS GLIMMIX procedure was employed to test the model in the Kilosa district and for all of Tanzania.

#### 2.3.3 Assessment of the Validation Data Collected by the Students

The validation data are also subject to some uncertainties due to differences in interpretation between individuals. Disagreement between interpreters is reported and discussed, since for most of the images, more than one observer evaluated the amount of cropland present. The disagreement was calculated as the difference between the minimum and maximum values that an image was assigned by the students; 0 denotes no difference up to a maximum value of 3 for the highest level of disagreement.

#### 2.3.4 Spatial Comparison of Cropland

Confusion matrices were produced that compare the validation data set against the four other data sets in four different classes/categories. The four categories were chosen based on the average amount of cropland in each 1 × 1 km scene that was reported by each data set, where <10% cropland was classified as “no cropland”, between 10% and 33% cropland was classified as “low” cropland, between 33% and 66% cropland was classified as “medium” cropland and higher than 66% was classified as “high” cropland. Note that 10% was used as a threshold for “no cropland” to reduce the noise that may occur due to the aggregation of the data sets to 1 km, particularly for data sets at a 30 m resolution.

Additionally, maps showing agreement between the validation data set and the four other data sets were produced for the Kilosa district. For these maps, a threshold of 50% cropland in a given scene was used to differentiate between “cropland” and “no cropland”. Finally, a map showing agreement between all data sets using the same definition of cropland/no cropland (50% threshold) was also produced. In all of these figures and tables, 22,190 frames were used to ensure that at least two students evaluated the same scene.

#### 2.3.5 Validation of Disagreeing Areas in the Kilosa District

Following the methodology of Fritz et al. [12], a random sample of 60 scenes in the Kilosa district was selected where all the global/regional products disagreed. The sample is 1% of the total number of disagreeing scenes, and since the average cropland variance across all four data sets is 0.86, this sample is deemed to be representative for the whole district with a confidence interval of approximately 15%. Three members of IIASA staff and two members of UMD staff with a background in remote sensing or geospatial sciences and considerable experience in image classification were chosen as experts. Each expert classified the 60 scenes using the Geo-Wiki interface. The results were then compared against all of the global products and the validation data set collected by the students as a further independent verification of the different data sets.

## 3 Results

### 3.1 Cropland Comparison across the Different Data Sets

**Figure 5:**
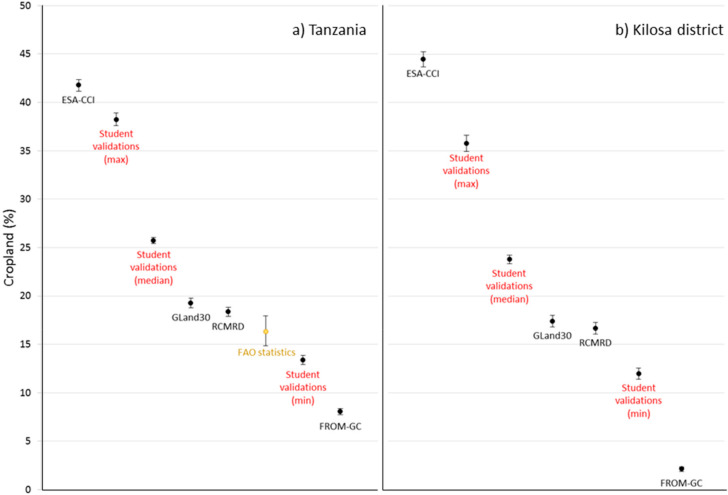
Average (median) cropland (%) with bars showing upper and lower 95% confidence limits for (**a**) Tanzania (N obs = 170,296, N unique scenes = 25,935) and (**b**) Kilosa district (N obs = 95,251, N unique scenes = 14,533), sorted from highest to lowest. FAO statistics for Tanzania are included as a reference showing the mean (in yellow), as well as the minimum and maximum values for the years 2008 to 2014.

The validation data set collected by the students is presented in the results as the average (median), minimum (min), and maximum (max) cropland as observed by the students. The comparison of cropland values from different sources in Tanzania and Kilosa follow the same order, i.e., ESA-CCI > Validation data set (max and median) > GlobeLand30 > RCMRD > Validation data set (min) > FROM-GC. The highest value shown by the ESA-CCI data set is around 44% cropland in Kilosa, with FROM-GC showing the lowest value also in Kilosa, ca. 2%. Figure 5 shows the adjusted medians for the generalized linear mixed models (Section 2.3.2) run for (a) Tanzania (N obs = 170,296, N unique scenes = 25,935) and (b) Kilosa district (N obs = 95,251, N unique scenes = 14,533) with bars showing upper and lower 95% confidence limits. All sources are significantly different from each other except for the data from GlobeLand30 and RCMRD in the Kilosa district.

The ESA-CCI data set shows 16% more cropland than the median of the Validation data set across Tanzania and is over 20% higher in Kilosa. The median of the Validation data set shows around 6% more cropland (ca. 26%) than GlobeLand30, and three times more cropland than the FROM-GC data set across Tanzania. These differences are similar for Kilosa but the median of the Validation data set is 11 times higher than FROM-GC.

For comparison, the FAO statistics have been added for Tanzania to Figure 5. The 2008 figure for area under cropland is based on data reported in an official country publication according to FAOSTAT but the figures for 2009 to 2014 are manual estimations from FAO. The closest match of these figures with the data sets is GlobeLand30 and RCMRD and they fall in between the minimum and the median estimates from the Validation data set.

### 3.2 The Disagreement within the Validation Data Set Collected by the Students

**Figure 6:**
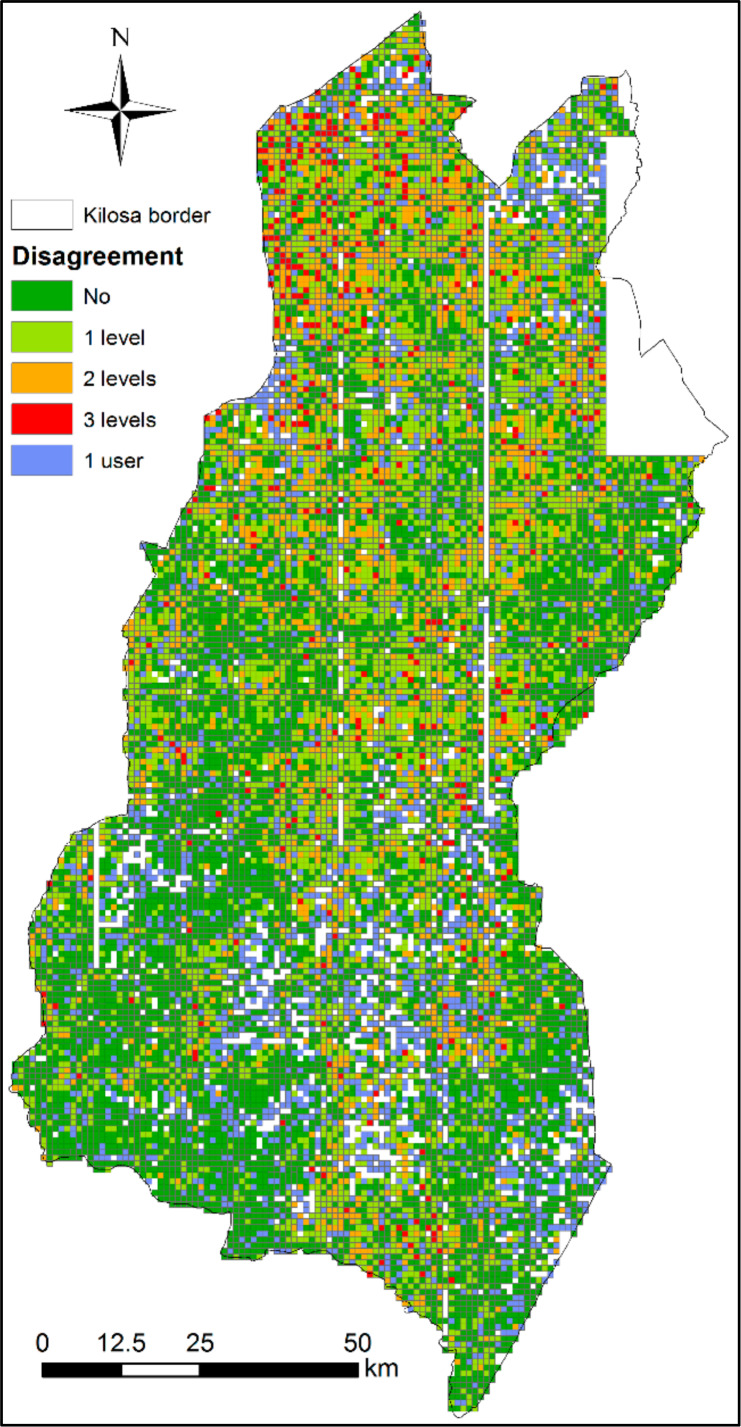
Cropland disagreement between the student interpreters for the Kilosa district, Tanzania.

Since the large majority of scenes had at least two interpretations from different people, several statistics could be obtained with regards to cropland coverage, with some of these being shown in Figure 5. Additionally, Figure 6 shows the disagreement between students spatially for each scene covering the Kilosa district. From the total number of scenes, 48% show complete agreement, 33% disagree by one level of cropland category, e.g., one user mentions low cropland and the other says no cropland or medium cropland. Additionally, 15% disagreed by two levels of cropland category, and complete disagreement (i.e., across three levels) occurred 4% of the time.

### 3.3 Disagreement between the Validation Data Set and the Cropland Data Sets

Tables 5–8 show confusion matrices with levels of cropland as detected by the students compared to each of the other data sets across Tanzania whereas Tables 9–12 show the same information specifically for the Kilosa district.

**Table 5:** Confusion matrix showing number of images classified as no cropland (<10%), low (10–33.3%), medium (33.3–66.6%) and high (>66.6%) cropland by the students compared to the ESA-CCI data set across Tanzania (N = 22,190). A total of 3745 scenes have only one interpretation and are not compared. The classification uses the average values from each data set. Users and producers (weighted) accuracy with 95% confidence intervals are shown. The overall weighted accuracy is 0.46 ± 0.01.

		Validation Data from the Students				
		No	Low	Mid	High	User Acc.
**ESA-CCI**	No	5508	2046	972	325	0.62 ± 0.01
	Low	986	552	405	187	0.26 ± 0.02
	Mid	928	632	610	410	0.24 ± 0.02
	High	1582	1573	2672	2802	0.32 ± 0.01
	Prod. Acc.	0.69 ± 0.01	0.10 ± 0.01	0.14 ± 0.01	0.70 ± 0.02

**Table 6:** Confusion matrix showing number of images classified as no cropland (<10%), low (10–33.3%), medium (33.3–66.6%) and high (>66.6%) cropland by the students compared to the GlobeLand30 data set across Tanzania (N = 22,190). A total of 3745 scenes have only one interpretation and are not compared. The classification uses the average values from each data set. Users and producers (weighted) accuracy with 95% confidence intervals are shown. The overall weighted accuracy is 0.47 ± 0.01.

		Validation Data from the Students				
		No	Low	Mid	High	User Acc.
**GlobeLand30**	No	8472	3839	2226	637	0.56 ± 0.01
	Low	261	471	712	323	0.27 ± 0.02
	Mid	138	263	770	629	0.43 ± 0.02
	High	133	230	951	2135	0.62 ± 0.01
	Prod. Acc.	0.68 ± 0.02	0.36 ± 0.02	0.34 ± 0.01	0.52 ± 0.02

**Table 7:** Confusion matrix showing number of images classified as no cropland (<10%), low (10–33.3%), medium (33.3–66.6%) and high (>66.6%) cropland by the students compared to the RCMRD data set across Tanzania (N = 22,190). A total of 3745 scenes have only one interpretation and are not compared. The classification uses the average values from each data set. Users and producers (weighted) accuracy with 95% confidence intervals are shown. The overall weighted accuracy is 0.43 ± 0.01.

		Validation Data from the Students				
		No	Low	Mid	High	User Acc.
**RCMRD**	No	7813	3541	2055	554	0.57 ± 0.01
	Low	767	713	1008	648	0.23 ± 0.01
	Mid	275	378	834	950	0.34 ± 0.02
	High	149	171	762	1572	0.59 ± 0.02
	Prod. Acc.	0.58 ± 0.01	0.33 ± 0.02	0.31 ± 0.01	0.48 ± 0.01

**Table 8:** Confusion matrix showing number of images classified as no cropland (<10%), low (10–33.3%), medium (33.3–66.6%) and high (>66.6%) cropland by the students compared to the FROM-GC data set across Tanzania (N = 22,190). A total of 3745 scenes have only one interpretation and are not compared. The classification uses the average values from each data set set. Users and producers (weighted) accuracy with 95% confidence intervals are shown. The overall weighted accuracy is 0.36 ± 0.01.

		Validation Data from the Students				
		No	Low	Mid	High	User Acc.
**FROM-GC**	No	8357	4187	3567	2437	0.45 ± 0.01
	Low	332	284	366	368	0.21 ± 0.02
	Mid	216	207	389	410	0.32 ± 0.03
	High	99	125	337	509	0.47 ± 0.03
	Prod. Acc.	0.47 ± 0.02	0.29 ± 0.02	0.29 ± 0.02	0.39 ± 0.02

**Table 9:** Confusion matrix showing number of images classified as no cropland (<10%), low (10–33.3%), medium (33.3–66.6%) and high (>66.6%) cropland by the students compared to the ESA-CCI data set for the Kilosa district (N = 12,373). A total of 2160 scenes have only one interpretation and are not compared. The classification uses the average values from each data set. Users and producers accuracy with 95% confidence intervals are shown. The overall accuracy is 0.42 ± 0.01.

		Validation Data from the Students				
		No	Low	Mid	High	User Acc.
**ESA-CCI**	No	2947	945	353	69	0.68 ± 0.01
	Low	698	376	216	62	0.28 ± 0.02
	Mid	655	426	369	181	0.23 ± 0.02
	High	980	993	1606	1497	0.29 ± 0.01
	Prod. Acc.	0.56 ± 0.01	0.14 ± 0.01	0.15 ± 0.01	0.83 ± 0.02

**Table 10:** Confusion matrix showing number of images classified as no cropland (<10%), low (10–33.3%), medium (33.3–66.6%) and high (>66.6%) cropland by the students compared to the GlobeLand30 data set for the Kilosa district (N = 12,373). A total of 2160 scenes have only one interpretation and are not compared. The classification uses the average values from each data set. Users and producers accuracy with 95% confidence intervals are shown. The overall accuracy is 0.54 ± 0.01.

		Validation Data from the Students				
		No	Low	Mid	High	User Acc.
**GlobeLand30**	No	4956	2172	1222	350	0.57 ± 0.01
	Low	168	285	411	140	0.28 ± 0.03
	Mid	84	151	426	318	0.44 ± 0.03
	High	72	132	485	1001	0.59 ± 0.02
	Prod. Acc.	0.94 ± 0.01	0.10 ± 0.01	0.17 ± 0.01	0.55 ± 0.02

**Table 11:** Confusion matrix showing number of images classified as no cropland (<10%), low (10–33.3%), medium (33.3–66.6%) and high (>66.6%) cropland by the students compared to the RCMRD data set for the Kilosa district (N = 12,373). A total of 2160 scenes have only one interpretation and are not compared. Users and producers accuracy with 95% confidence intervals are shown. The classification uses the average values from each data set. The overall accuracy is 0.50 ± 0.01.

		Validation Data from the Students				
		No	Low	Mid	High	User Acc.
**RCMRD**	No	4569	2033	1169	277	0.57 ± 0.01
	Low	476	413	558	325	0.23 ± 0.02
	Mid	163	203	442	472	0.35 ± 0.03
	High	72	91	375	735	0.58 ± 0.03
	Prod. Acc.	0.87 ± 0.01	0.15 ± 0.01	0.17 ± 0.01	0.41 ± 0.02

**Table 12:** Confusion matrix showing number of images classified as no cropland (<10%), low (10–33.3%), medium (33.3–66.6%) and high (>66.6%) cropland by the students compared to the FROM-GC data set for the Kilosa district (N = 12,373). A total of 2160 scenes have only one interpretation and are not compared. Users and producers accuracy with 95% confidence intervals are shown. The classification uses the average values from each data set. The overall accuracy is 0.44 ± 0.01.

		Validation Data from the Students				
		No	Low	Mid	High	User Acc.
**FROM-GC**	No	5215	2655	2338	1577	0.44 ± 0.01
	Low	35	52	92	93	0.19 ± 0.05
	Mid	25	17	77	78	0.39 ± 0.07
	High	5	16	37	61	0.51 ± 0.09
	Prod. Acc.	0.99 ± 0.01	0.02 ± 0.01	0.03 ± 0.01	0.03 ± 0.01

**Figure 7:**
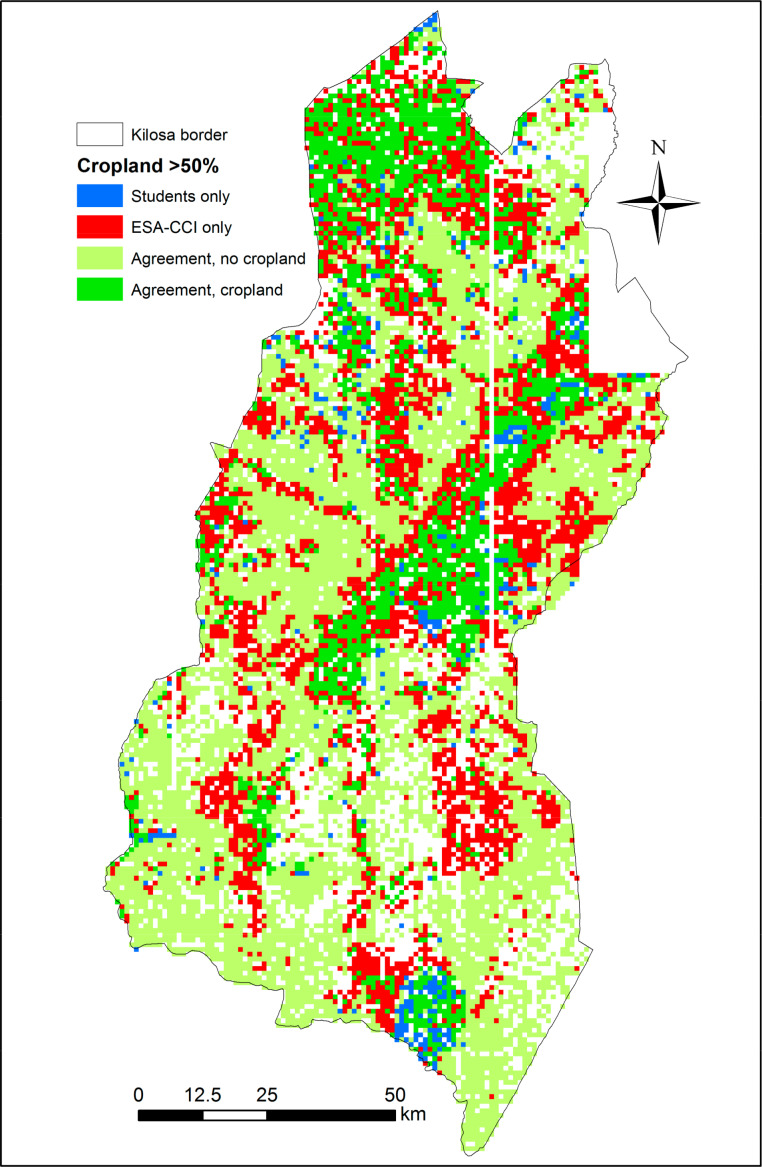
Disagreement between the student validation and ESA-CCI data sets in the Kilosa district where each scene is considered as cropland if it has an average of more than 50% cropland (N = 12,373). A total of 2160 scenes have only one student interpretation and are shown as blank spaces.

**Figure 8:**
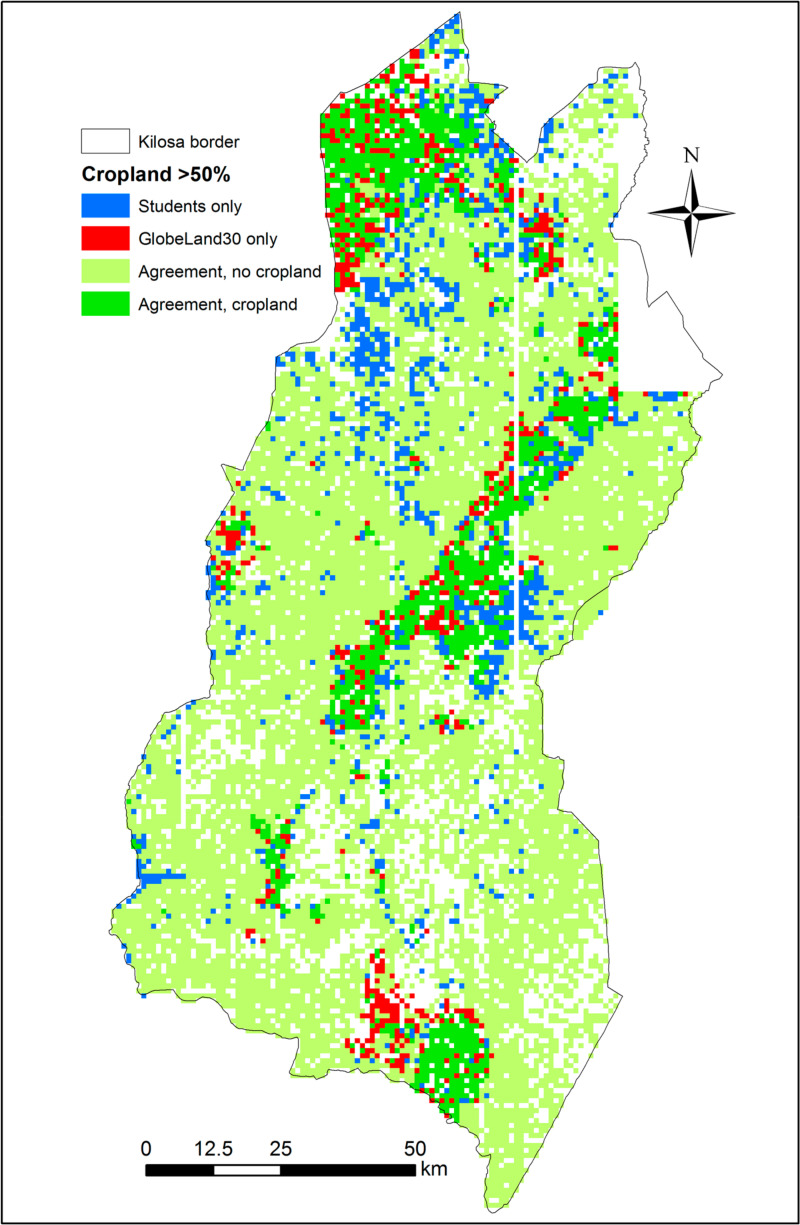
Disagreement between the student validation and GlobeLand30 data sets in the Kilosa district where each scene is considered as cropland if it has an average of more than 50% cropland (N = 12,373). A total of 2160 scenes have only one student interpretation and are shown as blank spaces.

**Figure 9:**
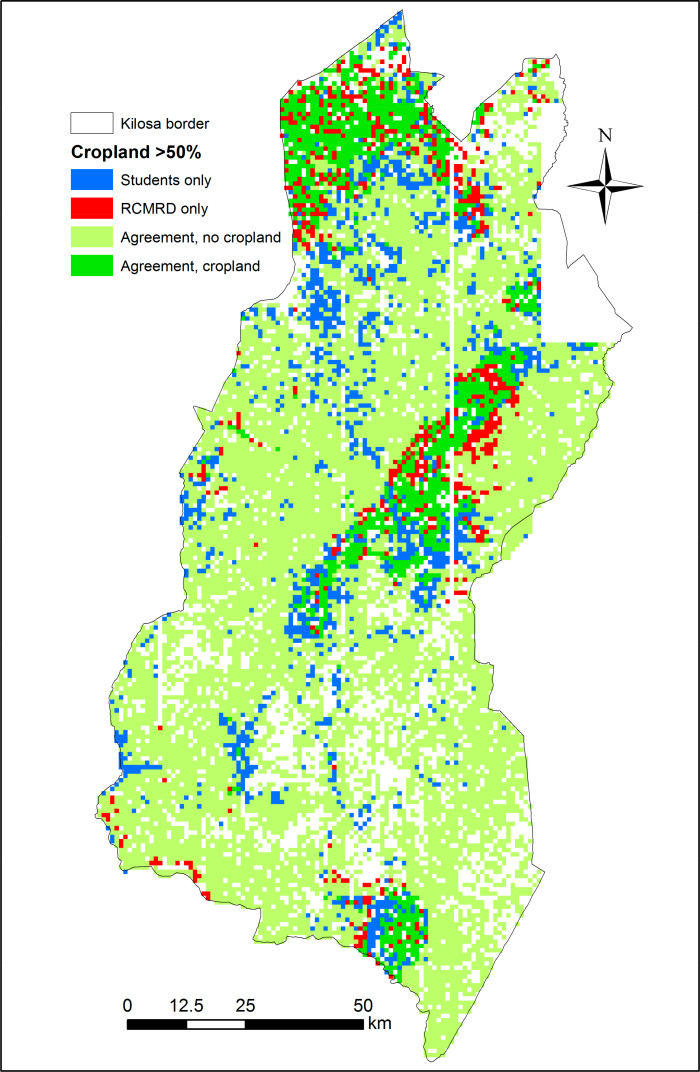
Disagreement between the student validation and RCMRD data sets in the Kilosa district where each scene is considered as cropland if it has an average of more than 50% cropland (N = 12,373). A total of 2160 scenes have only one student interpretation and are shown as blank spaces.

**Figure 10:**
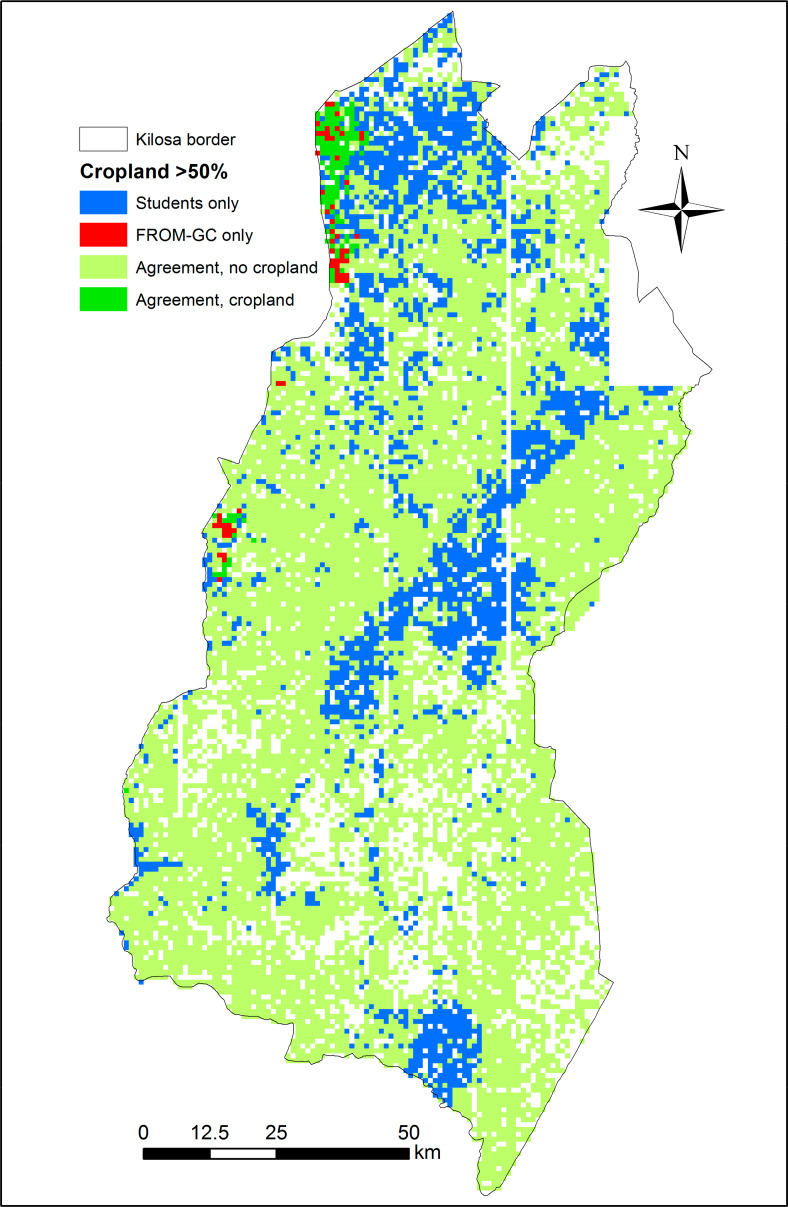
Disagreement between the student validation and FROM-GC data sets in the Kilosa district where each scene is considered as cropland if it has an average of more than 50% cropland (N = 12,373). A total of 2160 scenes have only one student interpretation and are shown as blank spaces.

Figures 7–10 show the spatial disagreement in cropland between the different data sets compared to the validation data set from the students.

**Figure 11:**
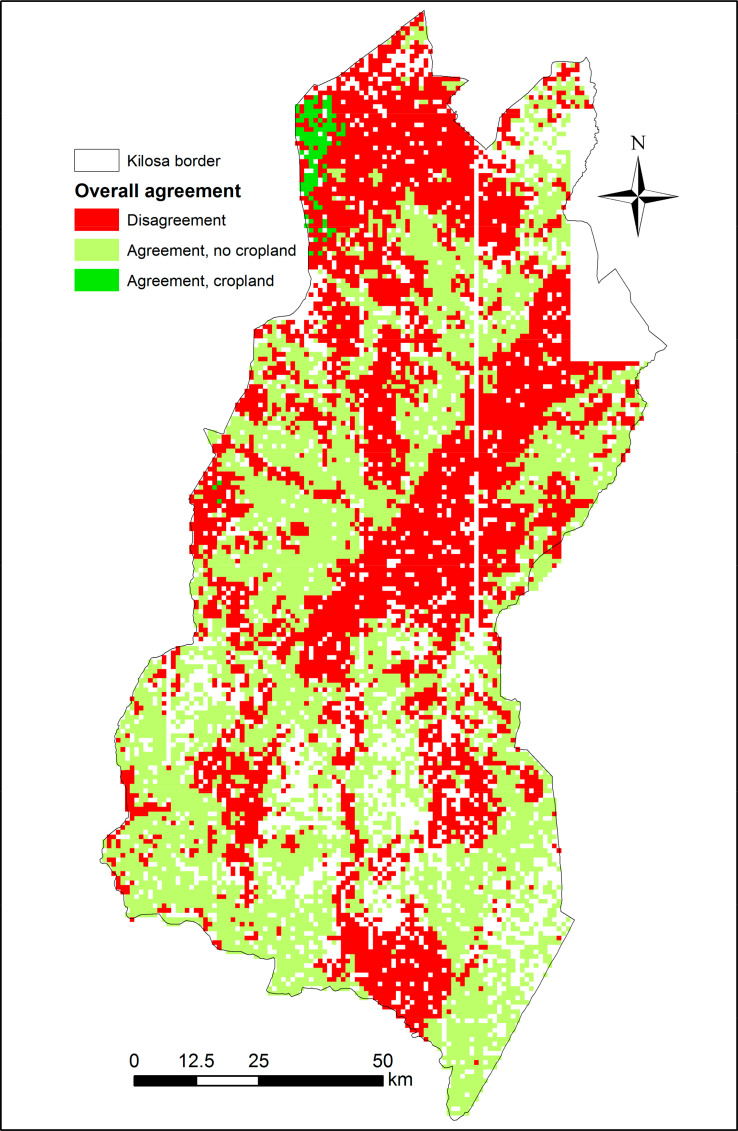
Overall agreement across all cropland data sets in the Kilosa district where a given scene is considered as cropland if it has an average of more than 50% cropland (N = 12,373). A total of 2160 scenes have only one student interpretation and are shown as blank spaces.

Finally, Figure 11 shows where all of the sources agree or disagree in the Kilosa district, with 52% of the scenes agreeing; of those, 98% show no cropland. A frame is classified as cropland when it has an average of 50% cropland or more.

### 3.4 Expert Verification of the Disagreement

Out of a total of 9817 frames covering Tanzania (omitting Kilosa), 53% agree (5182 frames). From those, 92% are classified as no cropland. A similar proportion of agreeing frames is preserved in Kilosa, where 51% of frames (6355 frames, shown in green, Figure 11) agree; 98% of those have no cropland.

In the disagreeing areas in Kilosa (6018 frames, shown in red, Figure 11), the proportion of frames classified as cropland are as follows: ESA-CCI 82%, validation data from the students 39%, GlobeLand30 34%, RCMRD 31%, and FROM-GC 3%. These proportions, except for FROM-GC with much larger amounts of cropland at 22%, are in line with the rest of the country, where the proportions of frames classified as cropland on disagreeing areas (4635 frames) are distributed as follows: ESA-CCI 70%, Validation data from the students 43%, GlobeLand30 38%, and RCMRD 34%.

**Figure 12:**
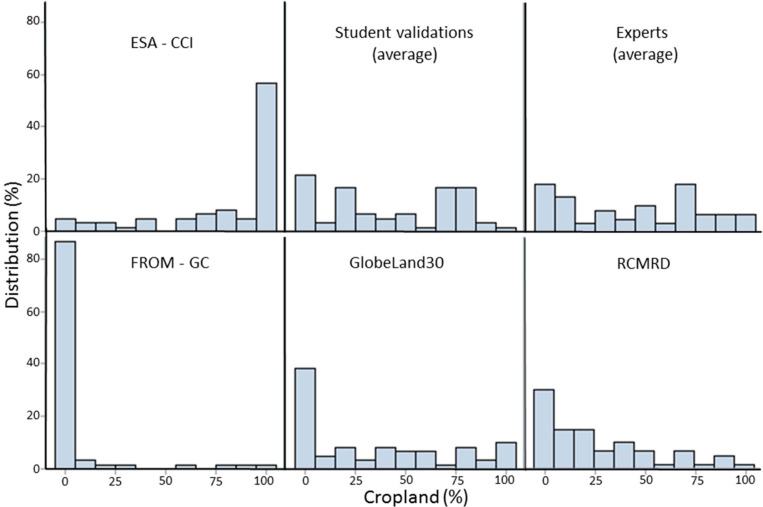
Histograms of cropland percentage distribution for all data sets in the selected 60 locations where disagreement was highest including the expert evaluations.

A confusion table comparing the student validations and the expert verification of a subsample of 60 disagreeing areas is shown in Table 13. The cropland distribution in the 60 evaluated locations is shown in Figure 12. The overall agreement between the two groups is 0.60, which is lower than that achieved when compared to the agreement between the students but this is to be expected since the disagreeing areas were harder to interpret. On a positive note, there was no confusion between the students and the experts in extreme cases, i.e., where the students said no cropland, the experts said high cropland and vice versa. Most of the confusion occurs within one class, e.g., between medium cropland (students) and high cropland (experts) while there are a few example of omission errors in identifying cropland by both experts and the students.

**Table 13:** Confusion matrix showing the number of images classified as no cropland, low, medium, and high cropland by the students compared to the expert classification on 60 locations where disagreement between the products was high. Users and producers accuracy with 95% confidence intervals are shown. The classification uses the average values from each data set. The overall accuracy is 0.60 ± 012.

		Expert Evaluations				
		No	Low	Mid	High	User Acc.
**Validation data from the students**	No	11	1	1	1	0.79 ± 0.22
	Low	4	3	4	1	0.40 ± 0.26
	Mid	0	0	6	5	0.44 ± 0.31
	High	0	3	4	16	0.69 ± 0.19
	Prod. Acc.	0.73 ± 0.17	0.43 ± 0.34	0.40 ± 0.20	0.70 ± 0.15

## 4 Discussion

**Figure 13:**
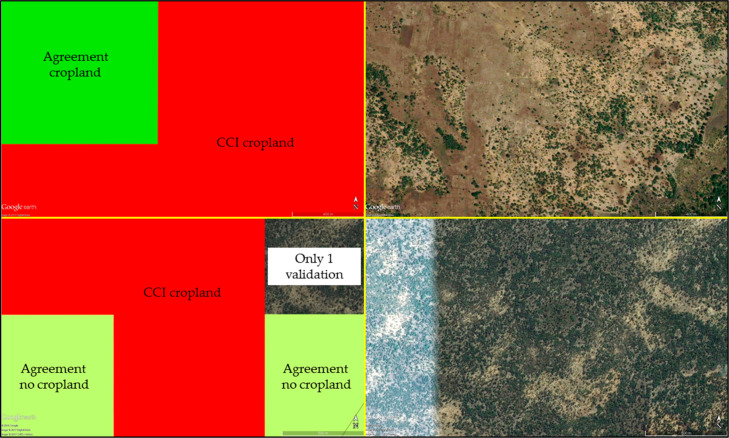
Visual inspection of very high resolution imagery using Google Earth showing two example sites where ESA-CCI classified grassland/shrubland as cropland.

The results show that there are considerable differences in the amount of cropland detected in the different data sets, with most cropland found in the ESA-CCI product and the least in the FROM-GC product. As shown in Tables 3 and 4, all products have comparable cropland classes, where only FROM-GC has some minor differences in the definition. In addition to including classes such as Crop-Bare and Crop-Greenhouse, FROM-GC does not include either rain-fed or irrigated tree crops. This might be one of the reasons why the total cropland is so low compared to the other products. The validation data collected by the students is closer to the ESA-CCI product only when considering the maximum values of the student validations. Note that we have not even considered the mosaic cropland classes, which would raise the amount of cropland found in ESA-CCI even more. The ESA-CCI product is produced using five years of satellite imagery (2008 to 2012) so it does actually better match the dates of the VHR imagery used in the validation, which also varies between 2005 and 2014. However, the majority of the VHR imagery used was from 2010 and later. FAO figures for Tanzania show that cropland increased by around 3.7% from 2008 to 2010. This number is small compared to the size of the differences between the different cropland products but could result in some underestimation in the validation data collected by the students. Similarly cropland increases after 2010 (16.7% between 2010 and 2012 and then stays stable after that according to FAO statistics) so there may be some overestimation in the validation data collected by the students. However, the ESA-CCI overestimates cropland compared to the validation data set, which itself may have some overestimation as outlined above, and hence the ESA-CCI clearly overestimates cropland. A visual inspection of areas where cropland is found in the ESA-CCI compared to VHR Google Earth imagery shows that these areas are mostly misclassified as grassland or shrubland, as shown in the examples in Figure 13.

The validation data set collected by the students is closest to GlobeLand30 and the RCMRD data sets when considering the median of the student interpretations. Both of these data sets have a considerable amount of manual checking built into their classification workflows so are not the product of only automatic classification algorithms. Fully automated approaches have led to either overestimation (ESA-CCI) or underestimation (FROM-GC) of cropland. As shown by Yu et al. [15], FROM-GC underestimates cropland by around 37% compared to FAO statistics in the region that they refer to as Middle Africa, which would include Tanzania. The reasons that the authors provide for this underestimation include the problems with obtaining cloud-free images in tropical areas; field sizes are small so there may be spectral mixing at 30 m resolution; and misclassification errors due to insufficient training samples, e.g., paddy rice being misclassified as water. The slight differences in cropland definitions as highlighted above may also be contributing to this underestimation.

Comparing the data sets to FAO statistics for Tanzania, the closest data sets are GlobeLand30 and the RCMRD product while the official statistics fall somewhere in between the minimum and the median of the validation data set collected by the students. Looking at the spatial agreements between these data sets and the validation data set collected by the students for Kilosa, the patterns are quite similar in that the main areas with cropland areas are in agreement. The student validation data set omits cropland in areas at the fringes of the existing cropland areas while there is evidence of cropland in the middle of Kilosa that is not picked up by either data set, which may indicate areas of cropland expansion identified in images after 2010. Hence either of these data sets has the potential to be used for agricultural monitoring purposes.

Another potential source of error could come from comparing data sets with different resolutions, which required aggregation of the data to match the 1 km grid cells of the validation data set. The aggregation may have introduced some artifacts, e.g., 300 m grid cells from ESA-CCI may have been split to fit the 1 km grid cells. Although the validation data set collected by the students is not error free, as clearly evidenced by the comparison between the students and the experts, such a data set does provide a valuable type of reality check for products derived from a top down remote sensing approach.

In terms of the robustness of the validation data, the analysis showed that there is greater than 80% agreement between user interpretations at the same location or that they differed by only one level of cropland category. The agreement with experts was lower, i.e., 60%, but this result was expected since the sample of 60 locations was chosen from areas of disagreement and hence were more difficult areas to interpret. However, most of the confusion was similarly between one level of cropland category and not at the extremes, e.g., where the students said no cropland and the experts said high cropland and vice versa. Although one level may seem like a large difference, the reasons for these findings may be because it is not always easy to interpret cropland from the VHR satellite images due to lack of color in some images, presence of cloud cover, the lack of clarity of features in the images, and images that are on the thresholds between low/medium and medium/high cropland. There were only a few examples of where either the students or the experts omitted cropland so this indicates that converting the data to binary cropland/non-cropland may produce a data set that can be used with reasonably high confidence since most of the confusion occurs in the amount of cropland identified. Considering that the exercise was undertaken wall-to-wall, it would be possible to derive a sufficiently large validation sample from the data set collected by the students.

Another possible use for such a data set would be in training future classifiers, where evidence of small fields can be readily recognized from the images. This is further backed up by a comparison of the student validation data with the other data sets. The ESA-CCI was missing cropland, 38% of the time compared to the student validation data, while these numbers are higher for the other products, i.e., 44% for GlobeLand30 and the RCMRD layer, and 55% for FROM-GC when considering Tanzania as a whole; similar results were obtained for Kilosa. However, there were also instances where the student validation data set showed no cropland and it was recorded in the other data sets. In most cases, the confusion is between no cropland and low cropland; the exception is ESA-CCI where there is confusion in both the low and high cropland classes.

Figure 11 provides an example of producing a hybrid map for Kilosa that can act as a cropland uncertainty layer. Thus, where all the products agree, then certainty is high that cropland exists at this location. Such products can provide users with more confidence, especially given the large variation in total cropland between the five different products. The individual disagreement layers can also provide guidance to the producers of the different products regarding where they could potentially sample for additional training data.

In terms of the technology used to collect the data, the offline Geo-Wiki client has real advantages over the regular Geo-Wiki tool. First, the images were directly obtained from Digital Globe so the precise dates of the imagery are known. This information is more difficult to obtain from Google Earth while Bing imagery is only available for one date so the image interpretation in Geo-Wiki is very much driven by the availability of the imagery. This problem is controlled for when using images directly from the provider. Secondly, no internet is required and the client is lightweight and fast so can be brought to locations where there are volunteers in the country itself, which can also draw upon the expertise of locals while teaching them about image interpretation. The disadvantages are that the image quality is sometimes poor or cloud covered and there is no possibility to zoom into the image as would be possible using an application like Google Earth. This may also have affected the quality of the resulting interpretations collected by the students as mentioned above. An additional consideration is the use of visually interpreted VHR imagery as a golden standard compared to classified Landsat or MERIS-scale imagery. It is certainly expected that VHR imagery provides more accurate results but future work should test this by using field data, possibly including unmanned aerial vehicle (UAV) imagery and WorldView3 satellite imagery with 30 cm spatial resolution.

## 5 Conclusions

In Africa, food security is highly dependent on smallholder, subsistence agriculture for supporting the majority of its people. Therefore, for national and global agricultural monitoring, spatially explicit data on the distribution of cropland is essential for assessing the risk and extent of crop failure early in the growing season and before harvest. Yet the results of this comparison have shown that despite the increase in map products available, there is still considerable disagreement between them, both in the total amount of cropland and in the spatial distribution. However, of the four data sets considered here, both GlobeLand30 and the RCMRD data sets could be useful for agricultural monitoring purposes.

The validation data collected by the students, although requiring more effort compared to automated top down approaches, can provide a source of training data for improving future cropland products, and where sufficiently large in amount, can be sampled for validation. Disagreement layers between the different products can also provide map producers with information about where additional training samples should be collected.
